# Brain structural changes in cannabis dependence: association with MAGL

**DOI:** 10.1038/s41380-019-0577-z

**Published:** 2019-11-06

**Authors:** Peter Manza, Kai Yuan, Ehsan Shokri-Kojori, Dardo Tomasi, Nora D. Volkow

**Affiliations:** 1 National Institute on Alcoholism and Alcohol Abuse, National Institutes of Health, Bethesda, MD USA; 2 School of Life Science and Technology, Xidian University, 710071 Xi’an, Shaanxi PR China; 3 National Institute on Drug Abuse, National Institutes of Health, Bethesda, MD USA

**Keywords:** Addiction, Neuroscience

## Abstract

Cannabis use is rising, yet there is poor understanding of biological processes that might link chronic cannabis use to brain structural abnormalities. To lend insight into this topic, we examined white matter microstructural integrity and gray matter cortical thickness/density differences between 89 individuals with cannabis dependence (CD) and 89 matched controls (64 males, 25 females in each group) from the Human Connectome Project. We tested whether cortical patterns for expression of genes relevant for cannabinoid signaling (from Allen Human Brain Atlas postmortem tissue) were associated with spatial patterns of cortical thickness/density differences in CD. CD had lower fractional anisotropy than controls in white matter bundles innervating posterior cingulate and parietal cortex, basal ganglia, and temporal cortex. The CD group also had significantly less gray matter thickness and density in precuneus, relative to controls. Sibling-pair analysis found support for causal and graded liability effects of cannabis on precuneus structure. Spatial patterns of gray matter differences in CD were significantly associated with regional differences in monoacylglycerol lipase (MAGL) expression in postmortem brain tissue, such that regions with higher MAGL expression (but not fatty-acid amide hydrolase or FAAH) were more vulnerable to cortical thinning. In sum, chronic cannabis use is associated with structural differences in white and gray matter, which was most prominent in precuneus and associated white matter tracts. Regions with high MAGL expression, and therefore with potentially physiologically restricted endogenous cannabinoid signaling, may be more vulnerable to the effects of chronic cannabis use on cortical thickness.

## Introduction

Cannabis use and dependence (CD) has risen in recent years [1], paralleling its legalization across the United States [2]. Although a wealth of evidence points to numerous adverse consequences of heavy cannabis consumption, including low quality of life [3], medical and psychiatric complications [4], cognitive impairment [5, 6], negative emotionality [7, 8], and psychiatric disorders [9], mechanisms mediating long-term effects of cannabis on brain structure remain unclear [10].

MRI studies have documented brain abnormalities in CD relative to healthy controls, although findings are mixed [11]. For instance, compared to controls, cannabis users show lower axonal connectivity [12] and fractional anisotropy [13] in corpus callosum and superior longitudinal fasciculi, but not conclusively [14]. In other tracts including forceps minor, findings are contradictory [14, 15]. The picture is similarly unclear for gray matter structure. Some studies reported lower volume or gray matter density in hippocampus, amygdala, and orbitofrontal cortex in cannabis users versus controls [16–18] whereas others report no differences [19–21]. Reports of group differences in cortical thickness have also been mixed [22–24]. Small sample sizes and failure to match control groups on relevant demographic and behavioral traits such as alcohol usage [21] have been limitations for many of these studies.

In addition, few studies have attempted to link endogenous cannabinoid signaling (ECS) to brain structural abnormalities in CD, which could help shed light on cannabis effects on specific brain structures, since whole brain volume does not appear to be affected in CD [21, 25]. Two key enzymes regulating ECS are fatty-acid amide hydrolase (FAAH) and monoacylglycerol lipase (MAGL), responsible for breaking down anandamide and 2-arachidonoylglycerol (2-AG), respectively, and thereby reduce local signaling from the two main endocannabinoids [26, 27]. These enzymes are of clinical interest, as work in rodents has shown that reducing FAAH expression through genetic or pharmacological manipulation is neuroprotective and confers resilience to anxiety [28–30]. Further, the FAAH inhibitor PF-04457845 reduced cannabis withdrawal severity and promoted abstinence in CD [31]. MAGL inhibitors are also being tested as pharmacotherapeutics [32]. Yet little is known about how regional brain expression of FAAH and MAGL influence brain structural changes in CD.

To help illuminate these issues, we examined white matter integrity and gray matter thickness and density in a relatively large cohort of individuals with CD and well-matched controls from the Human Connectome Project [33]. We tested the association between regional patterns of thickness/density differences in CD and FAAH/MAGL gene expression in postmortem tissue from the Allen Human Brain Atlas [34]. Since endogenous cannabinoids modulate excessive synaptic excitation and inhibition throughout the brain [35], we hypothesized that regions with low enzymatic expression (where ECS would be higher) would be less sensitive, whereas regions with high enzymatic expression (more precisely regulated ECS) would be more sensitive to chronic cannabis stimulation.

## Methods

### Participants

Participants provided written informed consent at Washington University in St. Louis. We identified 89 individuals meeting DSM-IV criteria for lifetime (current or prior) CD and without comorbid current or prior alcohol dependence, and a control group (CTL; *n* = 89) with <10 lifetime cannabis uses, carefully matched with the CD group on: age, sex, education, BMI, and alcohol usage [8, 36]. We attempted but could not match on tobacco usage, which was higher in the CD group (*p* < 0.001), and subsequent analyses were performed to ensure results were not driven by tobacco usage. For details see Supplementary Methods and Supplementary Tables 1 and 2.

### MRI image acquisition and preprocessing

Scans were collected using a custom-made Siemens Connectom Skyra scanner with a 32-channel head coil. T1- and T2-weighted anatomical scans were acquired at 0.7 mm isotropic resolution. Diffusion-weighted data were collected over six runs with three different shells of *b* = 1000, 2000, and 3000 s/mm^2^ (including 90 diffusion weighting directions and six *b* = 0 acquisitions interspersed throughout each run) [33].

### White matter microstructure: tract-based spatial statistics

Preprocessing of diffusion images by HCP investigators included *b*0 intensity normalization and correction for EPI distortion, eddy currents, subject motion, and gradient nonlinearities. Fractional anisotropy maps were calculated by fitting a tensor model at each voxel of the diffusion data. Participants’ fractional anisotropy images were nonlinearly registered to Montreal Neurological Institute (MNI) space and aligned to the skeletonized mean fractional anisotropy image of all participants.

### Gray matter structure: cortical thickness and gray matter density

Structural images were “minimally preprocessed” by HCP investigators through standardized pipelines and corrected for gradient nonlinearity-induced distortions, readout distortions, and intensity inhomogeneities, and aligned to MNI atlas. Then, images were processed through a customized version of Freesurfer. We used the cortical thickness values for each of 34 left-hemisphere cortical regions (parcels) in the Desikan−Killany parcellation [37], and conducted follow-up vertex-wise analysis, to assess if any regions showed group differences that were not observed at the parcel level. Only left-hemisphere values were used for comparability with the Allen Human Brain Atlas transcriptomic data, which only has left hemisphere data for all donor brains [38]. We also assessed if there was a similar pattern in group differences observed for gray matter density, as implemented in SPM12. In addition, we used the diffeomorphic anatomic registration through an exponentiated lie algebra algorithm (DARTEL) described previously [39], and applied 8 mm smoothing.

### Sibling-pair analysis: causal vs. predispositional effects of cannabis on brain structure

To address the question of whether high cannabis exposure causes brain structural deficits, or whether individuals were predisposed to have brain structural changes [40, 41], we took advantage of the multiple sibling pairs collected by the HCP. We compared sibling pairs concordant and discordant for heavy cannabis usage, as well as discordant for heavy or light cannabis exposure. Low cannabis exposure corresponded to criteria used for matched CTLs: <10 lifetime uses. For “high cannabis exposure”, due to limited sample size, we combined CD with individuals reporting >100 lifetime uses (without comorbid past or current alcohol dependence; for details, see Supplement). Based on this, we created four groups: individuals with a sibling (1) concordant for high cannabis exposure, (2) concordant for low exposure, (3) discordant with high exposure, and (4) discordant with low exposure. Our final sample was 820 sibling pairs from 347 families (41 pairs concordant for high exposure, 647 pairs concordant for low exposure, and 132 pairs discordant for exposure; for full details, see Supplementary Methods and Supplementary Table 3).

### FAAH/MAGL regional gene expression: Allen Human Brain Atlas

The Allen Human Brain Atlas contains gene expression data from six postmortem brains (age range 24–57; five male, one female; three Caucasian, two African-American, one Hispanic; http://help.brain-map.org/display/humanbrain/Documentation). We performed these recently established steps to map postmortem data into the same space as the MRI data [38]: (1) download and reannotate microarray probes; (2) exclude probes with poor signal due to nonspecific hybridization; (3) choose the single FAAH and MAGL probe with highest differential stability [42]; (4) assign probe samples to Desikan−Killany parcels based on distance along the cortical surface; (5) normalize data within each of the six brains using scaled robust sigmoid transformation [43]; (6) for each donor and each parcel, average all samples before averaging across all six donor brains (for more details, see Supplement).

### Statistical analyses

For DTI, we examined significant group differences in fractional anisotropy across the entire white matter skeleton using a two-sample *t* test with FSL’s *randomise* and 5000 permutations [44]. Significant clusters were identified by threshold-free cluster enhancement at a family-wise error (FWE)-corrected threshold of *p* < 0.05 [45].

For cortical thinning and gray matter density, our primary analyses were done in parcel space; we only performed voxelwise analysis for follow-up analysis. This is because parcel space is the recommended way to compare imaging data with the Allen Human Brain Atlas transcriptomic data, since probes were collected in different locations within parcels across individuals, and therefore exact assignment of probes to a voxel or vertex would limit the ability to aggregate transcriptomic data across postmortem donors [38]. Hence we examined group differences between CD and CTL in cortical thickness and density for each parcel through two-sample *t* tests, and used the false discovery rate Benjamini−Hochberg method to correct for multiple comparisons. For the exploratory whole-brain two-sample *t* tests, we used different thresholding procedures depending on the measure. For gray matter density, results were thresholded at *p* < 0.001 with a minimum cluster size of 600 voxels (2-mm isotropic, ~2 cm^3^) and a cluster-level correction for multiple comparisons of *p*_FWE_ < 0.05 in SPM [46, 47]. Since cortical thickness is in a two-dimensional sheet and does not follow the same assumptions as volume-based analysis, we used nonparametric permutation testing with FSL’s PALM software for multiple comparisons correction (*p*_FWE_ < 0.05; 5000 permutations). In addition, we tested the association between group differences in cortical thickness and gray matter density, to assess correspondence between them. We correlated the effect size of the group comparison (CD vs. CTL) for the two measures across all 34 brain regions. Because the HCP includes family members, we accounted for family structure using “exchangeability blocks” when making group comparisons in brain structure, where possible, to ensure that the null distribution was valid [48], as in our previous work [49].

For the sibling-pair analysis, similar to recent work [40], we used a linear mixed model to examine effects of cannabis exposure on regions showing significantly different cortical thickness between CD and CTL, with individuals nested inside of sibling pairs that were nested inside of families, using the *lmer* function [50] in R. In the final model, we controlled for all variables that were matched between CD and CTL groups: age, sex, BMI, composite alcohol usage (*z*-scored), composite tobacco usage (*z*-scored), education and depression and anxiety symptoms. The three primary contrasts of interest (“causal”, “graded liability”, and “predisposition” hypotheses [40]) were included as fixed effects; the contrast coding for each hypothesis is represented in Supplementary Table 4. If the “causal” hypothesis is supported, among discordant sibling pairs the individuals with high exposure would have significantly lower cortical thickness than their siblings with low exposure. If the “graded liability” hypothesis is supported, then the concordant siblings with high cannabis exposure would have lower cortical thickness than both the discordant high and low exposure individuals. If the “predisposition” hypothesis is supported, then discordant high and low exposure individuals would have similar cortical thickness to the concordant high exposure pairs, and all three of these groups would have lower thickness than the concordant low exposure group [40].

Then, we identified if adjacent white matter and gray matter regions showed common structural deficits (if any regions seemed to be a common locus for both gray and white matter deficits) in CD, and conducted correlations between gray and white matter structural deficits across participants at this locus. This was important because there is not sufficient spatial coverage of postmortem probes to link gene expression data to the small white matter bundles from the tract-based spatial analysis; we could only confidently link gene expression data to gray matter findings. We also tested if the association between white and gray matter integrity was stronger among CD than CTL by conducting a *z* test for difference in correlation slopes. If there was a significant association in CD but not CTL, this would provide evidence for a common deficit in CD affecting both white and gray matter integrity, rather than simply showing that adjacent white and gray matter regions tend to have common levels of integrity across the entire sample. We used Bonferroni-correction for multiple comparisons (cortical thickness and gray matter density (*p* < 0.05/2 = 0.025)).

We also assessed the correspondence between regional structural differences in CD with regional FAAH and MAGL gene expression. We conducted a spatial correlation between the gray matter structure group comparison effect sizes and the normalized FAAH/MAGL gene expression for each parcel showing structural changes in CD relative to CTL. To test for significant spatial correlations, we used permutation testing. Rows representing the gene expression values for each parcel were randomly permuted 5000 times, and the spatial correlation with gray matter structural group differences was rerun after each permutation. The significance threshold was the number of permutations with a stronger correlation than the observed correlation from the original data, divided by the total number of permutations. We further corrected for the four comparisons (FAAH and MAGL genes each correlated with cortical thickness and gray matter density) using Bonferroni-correction (*p* < 0.05/4 = 0.0125), which was a conservative approach given that cortical thickness and gray matter density were positively correlated across regions (Supplementary Fig. 1).

It is possible that any significant spatial correlations observed were due to low-order spatial gradients in gene expression or gray matter differences in CD [51]. In other words, even though the parcels of the Desikan−Killany atlas are thought to represent rather distinct cortical regions, there may be spatial dependencies between nearby regions that drive spatial correlations. If this were the case, then we would expect to see consistently inflated correlations between gray matter structural differences in CD and random genes expressed in brain tissue. To test if observed correlations were significantly stronger than those of random genes, we conducted spatial correlations between gray matter structural changes in CD and expression values from 5000 randomly selected genes in the database that passed the filtering procedure. Again, the significance threshold was the number of random genes with a stronger correlation than the correlation from the original data, divided by the total number of correlations.

Lastly, we performed several additional analyses to control for possible confounding effects. To test whether group differences (CD vs. CTL) in brain structure were driven by recent vs. past cannabis use, we assessed whether cortical thickness in left precuneus was significantly different among CD recent users (any use in last 12 months; *n* = 52) as compared to CD reporting no use in last 12 months (*n* = 37). We also tested whether there were differences among CD who tested positive (*n* = 33) vs. negative (*n* = 56) on a THC urine screen (Supplementary Fig. 2). To test whether group differences were driven by recent use or polydrug use, we reperformed our analyses with “THC urine screen” and “Times Used Illicit Drugs” each as a covariate. Finally, to examine if cannabinoid gene expression is associated with population characteristics of cortical thickness generally, we assessed whether MAGL and FAAH expression were spatially correlated with mean gray matter structure across the entire HCP cohort with structural MRI data (*n* = 1113). To test the significance of this spatial correlation, we used the same permutation testing approach stated above (5000 permutations). Code for these analyses is available upon individual request.

## Results

### White matter microstructure: tract-based spatial statistics

**Fig. 1:**
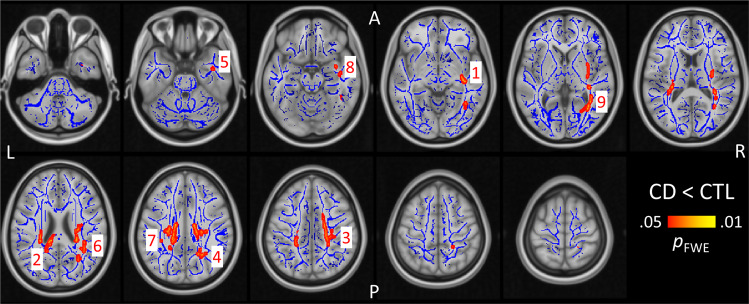
Group differences in white matter microstructure. Two-sample *t* test results showing regions with significantly lower fractional anisotropy in the CD group relative to CTL. Significant clusters are highlighted in red and are overlaid on the group mean white matter skeleton in blue. Numbers correspond to the “cluster number” column in Table 1. L left, R right, A anterior, P posterior, CD cannabis dependence, CTL controls

We compared whether white matter integrity, as indexed by fractional anisotropy, differed between CD and CTL along the white matter skeleton. Two-sample *t* tests revealed nine significant clusters where CD showed significantly lower fractional anisotropy than CTL (*p*_FWE_ < 0.05; Fig. 1; Table 1). These clusters included a right-lateralized set of tracts innervating the external capsule, posterior corpus callosum, inferior temporal cortex, posterior thalamic radiation, as well as bilateral superior longitudinal fasciculus. Among the CD group, fractional anisotropy did not correlate with tobacco usage in any cluster, even when uncorrected (|*r*|’s < 0.20, *p*’s > 0.05).

**Table 1:** Two-sample *t* test results showing regions with significantly lower fractional anisotropy in the CD group relative to controls

Region (JHU Atlas)	Cluster number	Voxels	*p*FWE	MNI coordinate		
				*X*	*Y*	*Z*
Sagittal striatum/external capsule R	1	1730	0.04	35	−15	−10
Splenium of corpus callosum	2	1418	0.033	−15	−34	27
Superior corona radiata R	3	356	0.047	21	−26	44
Posterior corona radiata R	4	331	0.044	25	−59	28
Inferior temporal R	5	119	0.048	39	−2	−35
Superior longitudinal fasciculus R	6	111	0.049	38	−48	20
Superior longitudinal fasciculus L	7	49	0.048	−33	−33	36
Uncinate fasciculus R	8	31	0.049	36	−1	−20
Posterior thalamic radiation R	9	3	0.05	29	−52	0

There were no significant results for the reverse contrast, i.e. CD > CTL. “Cluster number” refers to the labels provided in Fig. 1

*MNI* Montreal Neurological Institute, *R* right, *L* left

### Gray matter structure: cortical thickness and gray matter density

**Fig. 2:**
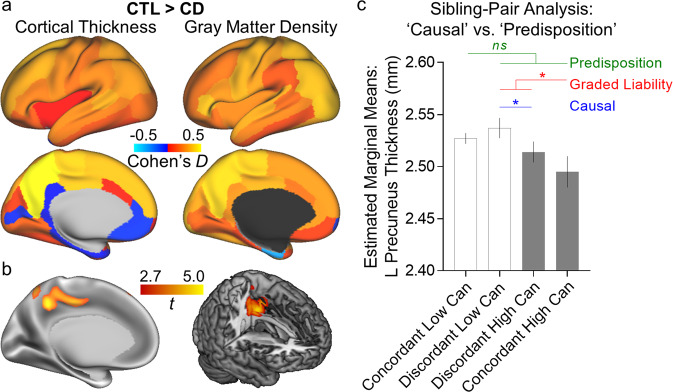
Group differences in gray matter structure. Two-sample *t* test results in **a** parcel space and **b** voxel/vertex-wise whole brain analysis. In parcel space, the precuneus was the only region showing significant group differences, after multiple comparisons correction. The whole-brain voxel/vertex-wise analyses showed a similar finding: one significant cluster in the precuneus emerged in each analysis where the CD group showed lower cortical thickness and gray matter density (results thresholded at *t* > 2.7, for visualization). **c** Sibling−pair analysis testing left precuneus cortical thickness in concordant and discordant pairs with low (<10 lifetime uses) vs. high (>100 uses or CD) exposure to cannabis. These data provide preliminary evidence for **a** causal effects of cannabis on precuneus cortical thickness, and **b** that precuneus cortical thickness deficits and heavy cannabis use might have common predispositional factors, with concordant high exposure pairs at the highest liability (that is, “graded liability”). Can cannabis. Error bars represent standard error of the mean. *p < 0.05

We assessed whether there were cortical thickness or gray matter density abnormalities in CD for all 34 cortical parcels in the left hemisphere (this was due to postmortem data being available only for left hemisphere). However, there was strong correspondence between left and right hemisphere gray matter structure: for the correlation across all subjects between each parcel on the left and right side: median *r*’s = 0.71/.77 for thickness/density, respectively. Although the CD group showed lower cortical thickness in most regions (27 out of 34; median effect size Cohen’s *D* = 0.17), the precuneus was the only region that remained significant after correction for multiple comparisons (*t*_(176)_ = 3.41, *p*_FDR_ = 0.028). Variance of precuneus thickness was not significantly different between the groups (*F* test; *F*_(88,88)_ = 1.029; *p* = 0.893). The CD group showed lower gray matter density in most regions compared to CTL (32 out of 34; median Cohen’s *D* = 0.26), again including a significant effect in precuneus (*t*_(176)_ = 2.81, *p* = 0.026; Fig. 2a). Follow-up whole-brain (voxelwise) analyses for cortical thickness and gray matter density each revealed a single significant cluster in the precuneus: center of mass MNI coordinates: *x* = 2, *y* = −24, *z* = 36; peak T-score = 4.7; cluster volume = 6.7 mL; Fig. 2b. These effects were not significantly correlated with tobacco usage in the CD group: for cortical thickness, *r*_(87)_ = −0.06, *p* = 0.576; for gray matter density, *r*_(87)_ = −0.18, *p* = 0.091. These findings were also not altered when including “THC urine screen” and “Times Used Illicit Drugs” as covariates (Supplement). There were no regions in which the CD group showed significantly higher thickness or density than CTL. There was a positive association between regional group differences in cortical thickness and in gray matter density (*r*_(32)_ = 0.53, *p* = 0.001) (Supplementary Fig. 1).

### Sibling-pair analysis: causal vs. predispositional effects of cannabis on brain structure

We examined sibling pairs of the HCP dataset and used a linear mixed model approach to examine precuneus cortical thickness differences between sibling pairs concordant or discordant for high or low cannabis exposure. The results provide some support for the “causal” and “graded liability” hypotheses, but not the “predispositional” hypothesis: “Causal” *t* = −2.130; *p* = 0.033; “Graded Liability” *t* = −2.039; *p* = 0.041; “Predispositional” *t* = 1.38; *p* = 0.168; Fig. 2c; Supplementary Table 5.

### Association between gray matter and white matter structural deficits in cannabis users

**Fig. 3:**
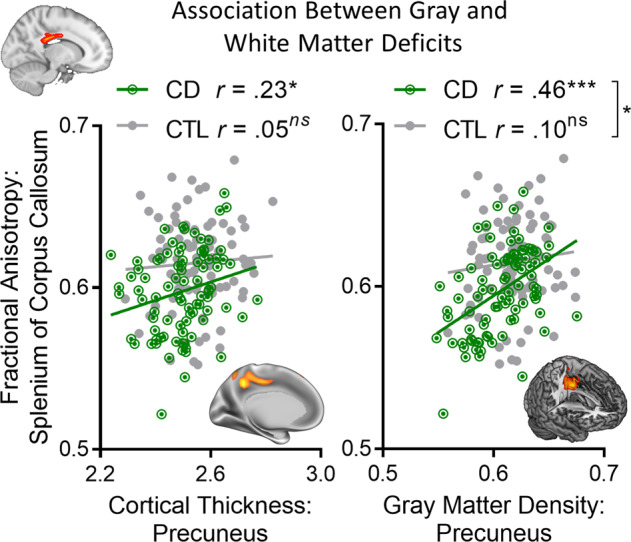
Association between gray matter and white matter deficits in cannabis users. Across individuals with cannabis dependence (CD), the white matter integrity of the splenium of corpus callosum (inset, top left) was positively associated with cortical thickness (left) and gray matter density (right) of the precuneus. However, no such association was observed in the CTL group, suggesting that this association may be specifically related to a common structural deficit observed in CD, and not simply due to the spatial proximity of corpus callosum and precuneus. The difference in slopes between groups for the association of fractional anisotropy and gray matter density was significant (*z* *=* 2.56; *p* = 0.011). Note: *p < 0.05; ***p < 0.001; ns nonsignificant

Since the precuneus emerged as the only region with gray matter structural deficits in both thickness and density in CD, we examined whether any neighboring white matter regions also showed deficits. The left splenium (posterior dorsal portion) of the corpus callosum emerged as the closest region with white matter deficits adjacent to the precuneus. As hypothesized, across CD participants, fractional anisotropy of the splenium was positively correlated with cortical thickness (*r* = 0.23, *p* = 0.030) and gray matter density (*r* = 0.46, *p* < 0.001) of the precuneus; no such association was observed in CTL (*r*’s = 0.05 and 0.10; *p*’s > 0.05 for thickness and density, respectively). The difference in correlation slopes between CD and CTL for the association of fractional anisotropy with gray matter density was significant (*z* = 2.56, *p* = 0.011; Fig. 3).

### Regional association between gray matter structure and FAAH/MAGL gene expression

**Fig. 4:**
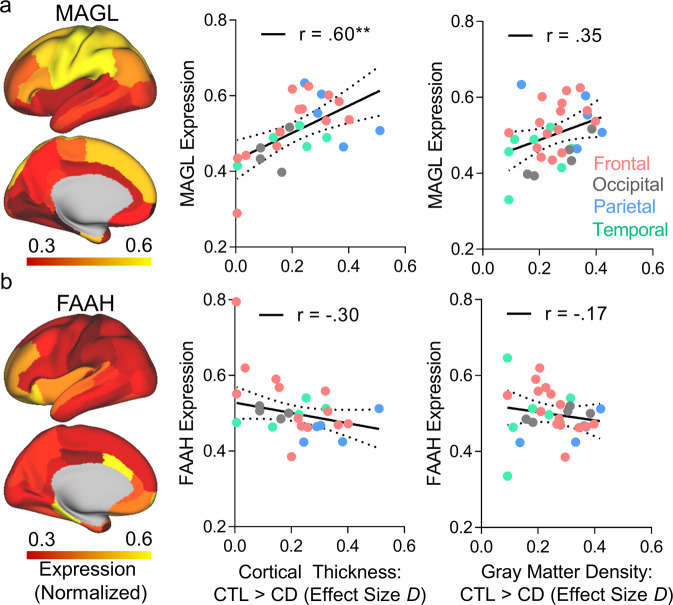
Association between cannabinoid enzyme gene expression and gray matter group structural differences. **a** Left: Regional distribution of MAGL gene expression across regions of the left cortical hemisphere (postmortem tissue); values were normalized within each of the six individual donor brains before averaging across all individuals. Center: spatial correlation between MAGL expression and group differences in cortical thickness, where higher numbers indicate thinner cortices in the CD group relative to controls (**p_FWE_ = 0.012). Right: spatial correlation between MAGL expression and group differences in gray matter density. **b** The bottom panel depicts the corresponding analyses for FAAH gene expression, in which we did not observe a significant association with gray matter structural group differences. The solid lines indicate the lines of best fit, and the dashed lines indicate the 95% confidence intervals

We then tested the regional correspondence between degree of cortical thickness/density deficits in CD and FAAH/MAGL gene expression. The spatial correlations revealed a significant positive association between cortical thinning in CD and MAGL expression, such that parcels with higher levels of cortical thinning (*n* = 27) had higher MAGL expression (*r*_(25)_ = 0.60, *p*_*FWE*_ = 0.012; Fig. 4). When examining gray matter density differences, the association with MAGL was in the same direction but significant at an uncorrected threshold only (*r*_(30)_ = 0.35, *p* *=* 0.049). The correlations between FAAH expression and cortical thinning (*r*_(25)_ = −0.30; uncorrected *p* = 0.104) and gray matter density differences (*r*_(30)_ = −0.17, uncorrected *p* = 0.368) were not significant.

The significant association between cortical thinning in CD and MAGL expression did not appear to be due to spatial dependencies between neighboring brain regions, which would cause systematically inflated correlations among randomly selected genes, whereas the correlation with MAGL was significantly higher than that with 5000 randomly selected genes (*p* = 0.013). In addition, mean cortical thickness of the entire HCP population with Freesurfer data (*n* = 1113) was not significantly associated with MAGL (*r*_(32)_ = −0.10, *p* = 0.565) or FAAH (*r*_(32)_ = 0.25, *p* = 0.157) expression.

## Discussion

CD individuals compared to matched CTL had lower white matter integrity and gray matter thickness/density, particularly in precuneus. Across the cortex, the pattern of gray matter differences showed a positive spatial correlation with regional expression of MAGL, the enzyme that breaks down the most prominent brain endocannabinoid, 2-AG. These data suggest that regions with a more precise regulation of ECS due to greater MAGL degradation of 2-AG may be the most vulnerable to adverse cannabis effects on brain structure.

### Cannabis dependence and white matter structural integrity

The CD group showed lower fractional anisotropy, a measure of white matter structural integrity, than CTL in several regions innervating amygdala/hippocampus, basal ganglia, and medial posterior cortical regions including precuneus. These data are consistent with findings of impaired axonal connectivity in heavy long-term cannabis users in tracts innervating the right hippocampus, precuneus, and posterior corpus callosum [12]. Our results also agree with one of the few longitudinal studies of chronic cannabis use, that showed reduced growth in fractional anisotropy in central/parietal superior longitudinal fasciculus and posterior corpus callosum in college-aged cannabis users over a 2-year period [13]. Though we did not observe significant effects in frontal white matter bundles, findings from prior studies have been inconsistent [14, 15]. Based on data in rodents that certain tracts like the corpus callosum have particularly high cannabinoid receptor expression during development, some have theorized that these tracts are especially vulnerable to cannabis exposure during adolescence [12], and small retrospective studies examining age of cannabis use onset tend to support this [52].

### Cannabis dependence and gray matter thickness/density

We also observed that, although the CD group showed lower gray matter thickness and density in most cortical regions, the size of these effects was generally small, with only the precuneus showing a significant group difference after multiple comparison correction. Follow-up whole-brain voxelwise analyses confirmed the specificity of the effect in precuneus. Our results are consistent with findings of gray matter density loss in precuneus in young adult cannabis users [53]. However, in late adolescence, cannabis use may be associated with increased cortical thickness [54] and volume in medial parietal cortex [25], which one study reported after only one or two uses [55]. Cortical gray matter size peaks by age 14, and thinning/volume loss in later adolescence is an important feature of development that may relate to synapse elimination [56]. It remains unclear if increased precuneus size in adolescence may eventually precipitate precuneus thinning/volume loss in adulthood, and how chronic cannabis use shapes this trajectory [22]. Brain structural deficits may be specific to heavy/dependent cannabis users, however, as earlier work on a smaller subset of the HCP data observed no differences in cortical size based on recreational cannabis use [36]. Another study using HCP data conducted a sibling-pair analysis and concluded that subcortical volume deficits were not caused by cannabis exposure, but rather that they were likely due to predispositional factors [40]. However, that study considered even one lifetime use of cannabis to be sufficient for inclusion in the cannabis group; here we examined individuals with a much more extensive history of cannabis use (>100 lifetime uses or CD) and found some evidence for both causal effects and graded liability. We also observed a strong correspondence for the effects of CD on thickness and density in precuneus, which were associated with nearby corpus callosum white matter integrity; this association was not present in CTL. This suggests that structural deficits reflect a common source of variation specific to cannabis use, and not due to spatial proximity of corpus callosum and precuneus.

### Cortical thickness differences in CD: association with MAGL expression

Finally, we observed that regions with higher expression of MAGL tended to show greater cortical thickness deficits in CD relative to CTL. MAGL is responsible for metabolizing up to 85% of 2-AG, the predominant endocannabinoid in brain [27]. Our finding follows a recent study in adolescents suggesting that increases in regional gray matter density from occasional cannabis use were positively correlated with brain CB1R expression [55]. Here we focused instead on two genes (*MAGL* and *FAAH*) that encode for the enzymes that degrade the main endocannabinoids (2-AG and anandamide, respectively) in the brain, since this is the primary mechanism for regulating ECS [26, 27]. Moreover, FAAH and MAGL have emerged as promising therapeutic targets for cannabis addiction [31, 32]. Our results suggest that brain regions with high MAGL expression, and therefore greater temporal restriction of 2-AG availability [27], are the most vulnerable to cortical thinning in CD. In rodent models 2-AG protects against neuronal loss following traumatic brain injury [57], and CB1R are necessary for protection against excitotoxic cell death [58, 59]. It is plausible therefore that the combination of downregulation of CB1R in CD [60], and low levels of synaptic 2-AG in brain regions with high MAGL expression, renders them more vulnerable to cortical thinning in adulthood. However, the precise mechanism behind cortical thinning in CD remains unclear. Note also that cortical downregulation of CB1R in cannabis users partially recovers after one month of abstinence [61, 62]. Therefore, it will be important to address whether understimulation or downregulation of CB1R precedes cortical thinning, or vice versa, and if either of these effects recovers with prolonged abstinence.

We had predicted that cannabis-related changes in cortical thickness would be associated with expression of FAAH and MAGL in brain, and while our findings provided support for MAGL we did not observe it for FAAH. This may reflect distinct brain concentration and role/functions of these enzymes [63, 64]. Indeed, the concentration of 2-AG in brain (nanomoles/gram) is much higher than for anandamide (pmol/gram) [65]. Their function also differs; 2-AG is released postsynaptically, acts on presynaptic CB1R to suppress neurotransmitter release [66], and supports depolarization-induced suppression of inhibition and excitation in most brain regions [67]. Anandamide in contrast might antagonize 2-AG via its partial agonist effects at CB1R [68]. MAGL may be particularly related to cortical thinning through its regulation of 2-AG, which has a greater involvement in synaptic plasticity than FAAH-regulated anandamide [69, 70]. Further, MAGL inhibitors increased glial-derived neurotrophic factors and prevented neurodegeneration in a mouse model of Parkinson’s disease, but FAAH inhibition did not [71]. These data, together with the predominance of 2-AG in cortex relative to anandamide, suggest that MAGL may be an important target for understanding cortical thinning in CD.

### Limitations

Limitations include: (1) This study is cross-sectional and although the sibling-pair analysis provided preliminary evidence that cortical thickness deficits may be caused by heavy cannabis exposure; our findings are nevertheless correlational. Follow-up studies with a larger monozygotic twin pair sample and longitudinal assessments will be critical to determine causality; (2) HCP has limited information on cannabis use, including THC content, frequency and patterns of consumption so we could not assess if differences were specific to users of higher doses. Further, many CD individuals had a negative THC urine screen and/or reported no cannabis use in the past 12 months. Therefore, our results pertain to lifetime history of use, and future work is needed to understand acute effects; (3) our analyses linking gene expression to cortical thickness differences were restricted to the left hemisphere due to coverage in the Allen Human Brain Atlas; though PET studies of FAAH [72] and MAGL distribution [73] suggest considerable bilaterality, future studies should assess right cortical hemisphere and subcortical regions; and (4) we could not link postmortem gene expression to white matter changes because of spatial limitations.

In summary, individuals with CD showed lower white matter integrity and gray matter thickness/density than well-matched controls, and the precuneus was the most affected region. Cortical thickness deficits in CD were positively associated with MAGL expression. Future studies could investigate whether MAGL inhibitors, which are being developed for treatment of CD, may restore these alterations.

## Supplementary information

Supplement
