# Supplementary material for: Brain structural changes in cannabis dependence: association with MAGL
Source: Mol Psychiatry. 2019 Nov 6;25(12):3256–66. doi: 10.1038/s41380-019-0577-z (PMC7200265; doi:10.1038/s41380-019-0577-z)

**Supplementary Material**

**Methods**

*Participants*

The participants included provided written informed consent at Washington University in St. Louis after receiving a complete description of procedures. We used all participants with complete imaging and behavioral data (*n* = 1003; aged 22-35) from the young adult HCP final release. From this population, we obtained data from 89 individuals meeting DSM-IV criteria for cannabis dependence (CD) and without comorbid alcohol dependence, based on an interview with a clinical research specialist. For inclusion as CD, individuals had to meet at least three of the following criteria: (1) tolerance to cannabis; (2) using cannabis in larger amounts or over a longer period than intended; (3) inability to cut down or reduce cannabis use; (4) spending large amounts of time to obtain, use, or recover from the effects of cannabis; (5) giving up important social, occupational, or recreational activities in favor of using cannabis; (6) continued use of cannabis despite its adverse consequences.

Recent work has indicated that it is critical in studies of CD to select a well-matched control group^1^. For this purpose, we identified a subset of participants (n = 562) with self-reported ≤ 10 lifetime uses of cannabis. Next we used the matchControls function in R (library e1071)^2^ to identify a control group (n = 86) from these participants (n = 562) that was matched with the CD group on the following variables: age, sex, education, BMI, and alcohol and tobacco usage. Tobacco/alcohol usage composite was calculated as done in recent studies using HCP data; see^3,4^. All indicated variables were matched between the two groups (*p*’s > .30) except tobacco usage, which was lower than in the CD group (*p* < .001). Subsequent analyses were performed to ensure results were not driven by tobacco usage. Demographics and group characteristics are presented in **Supplementary Tables 1 and 2**.

*FAAH/MAGL regional gene expression: Allen Human Brain Atlas*

To analyze regional patterns of gene expression, we followed recently-established procedures^5^: first, transcriptional expression data from microarray probes representing roughly 20,000 genes in over 3700 different brain regions were downloaded from the Allen Human Brain Atlas database, and probe information was updated using re-annotation software. Next, we excluded probes with poor signal due to non-specific hybridization, where signal did not exceed background intensity in at least 50% of all probes in all subjects. We chose the most representative probe for each gene (FAAH and MAGL) based on which probe had the highest differential stability; that is, the probe with the most consistent spatial pattern across the six donor brains^6^. For each of the six individual donor brains, we assigned each left-hemisphere cortical sample to one of the 34 Freesurfer *aparc* parcels in native space: samples were assigned if they fell within a surface-based distance of 2mm of any coordinate within one of the Desikan-Killany parcels. Consequently, expression values were normalized within each of the six individual brains using a scaled robust sigmoid transformation, which is resilient to outliers and produces equivalent normalization scaling for all donor brains^7^. Finally, for each donor brain and each parcel separately, we averaged all samples to create a mean normalized expression value, before averaging across all six donor brains. This procedure ensures that each donor has an equal contribution to the final expression value for a parcel, even if donors have differing number of probes that fall within a brain region.

*Analysis of Frequent Cannabis Users without CD*

It is possible that individuals with high exposure to cannabis, even if they do not meet criteria for CD, may have brain structural deficits relative to CTL. To examine this, we identified a third group: individuals who used cannabis >100 times but do not have symptoms of CD (without current or past AUD; n=80; this group was matched with CTL on all variables except sex, as the >100 use group had a greater proportion of females [35/80; 44%] than the CTL group [25/89; 28%]). With this third group (‘>100 use’ group) we sought to identify whether brain structural deficits are related to high cannabis exposure generally, or if they seem to be restricted to CD.

*Sibling Pair Analysis: Causal vs. Predispositional Effects of Cannabis on Brain Structure*

An important difference between our studies is that, whereas Pagliaccio et al.^8^ examined whether *any* exposure to cannabis (i.e., 1 or more lifetime uses) might result in volume deficits, we hypothesized that structural deficits would only emerge with many lifetime uses, as reflected in our decision to study the CD group. However, because using only the CD group greatly reduces the number of possible sibling pairs discordant for cannabis exposure, and because the ‘>100 use’ group showed generally similar results to the CD group, we combined the CD and ‘>100 use’ groups into a “high cannabis exposure” group for this analysis. To determine individuals with “low cannabis exposure”, we maintained the cannabis exposure criteria that we used for CTL: < 10 lifetime uses. Out of 1093 possible sibling pairs in the HCP, we excluded pairs for the following reasons: half-siblings, which can confound interpretation^9^ (4 pairs); individuals without Freesurfer data that passed quality control (135 pairs); and individuals that did not meet criteria for heavy cannabis use, but still had >10 lifetime uses (i.e., 11-99 lifetime uses; 134 pairs). After applying these criteria, our final sample was 820 sibling pairs from 347 families (41 pairs concordant for high exposure, 647 pairs concordant for low exposure, and 132 pairs discordant for exposure; for full details, see **Supplementary Table 3**). Similar to Pagliaccio et al., we used a linear mixed model to examine the effects of cannabis exposure on Left Precuneus Cortical Thickness, with individuals nested inside of sibling pairs that were nested inside of families, using the *lmer* function^10^ in R.

Importantly, we did not remove opposite-sex pairs discordant for cannabis use from our analysis, unlike the Pagliaccio et al. study^8^. This is because the prior study examined subcortical volumes, which systematically differ between males and females; however in the current analysis we examined cortical thickness, which does not show statistically significant sex effects for the left precuneus region of interest (*t*_(1111)_ = -0.473, *p* = .636). In fact, equivalence testing^11^ suggested that, even with the large sample of the HCP (507 males and 606 females with usable Freesurfer data), which is powered to detect very small effects (given α = .05 and 1-β = .80, Cohen’s *D* = .169, or the equivalent of .019 mm differences in cortical thickness), we observed that sex effects are statistically equivalent to zero (*t*_(1111)_ = 2.335, *p* = .009). Therefore, to improve our limited sample size, we retained all opposite-sex pairs and included sex as a covariate in the analysis.

In the final model, we controlled for all variables that were used to match the CD and CTL groups in the main manuscript: Age, Sex, BMI, Composite Alcohol Usage (*z*-scored), Composite Tobacco Usage (*z*-scored), Income, Education, Depression symptoms, and Anxiety symptoms. The three primary contrasts of interest are represented in **Supplementary Table 4**. Briefly, the individuals are divided into four groups: individuals with a sibling 1) concordant for high cannabis exposure, 2) concordant for low exposure, 3) discordant with high exposure, and 4) discordant with low exposure. If the ‘causal’ hypothesis is supported, among discordant sibling pairs the individuals with high exposure would have significantly lower precuneus thickness than their counterpart with low exposure. If the ‘graded liability’ hypothesis is supported, then the concordant siblings with high cannabis exposure would have lower cortical thickness than both the discordant high and low exposure individuals. If the ‘predisposition’ hypothesis is supported, then discordant high and low exposure individuals would have similar cortical thickness to the concordant high exposure pairs, and all three of these groups would have lower thickness than the concordant low exposure group^8^.

**Results**

*Control Analyses: THC urinalysis and Illicit Drug Use*

When including THC urine screen results as a covariate in the original cortical thickness group analysis, the CD group still shows significantly lower thickness of the left precuneus than CTL (*t*_(176)_ = 2.67, *p* = .008). Further, the spatial pattern of group differences across the cortex was highly correlated between the primary analysis and the analysis controlling for THC urine status (*r*_(32)_ = .96). Thus, the data do not support evidence of acute negative effects on brain structural integrity with cannabis use (relative to past heavy cannabis use).

We also included the “Times Used Illicit Drugs” as a covariate in the same primary analysis, and the results hold: The CD group shows significantly lower cortical thickness (*t*_(176)_ = 3.88, *p* = 1.4 x 10^-4^) of the left precuneus than CTL. The spatial pattern of group differences across the cortex was highly correlated between the primary analysis and the analysis controlling for illicit drug use (*r*_(32)_ = .80). Therefore, group differences do not appear to be driven by history of use of other illicit drugs aside from cannabis (**Supplementary Figure 2)**.

*Analysis of Frequent Cannabis Users without CD*

We examined if frequent cannabis users (>100 lifetime uses) without symptoms of CD would show cortical thickness deficits relative to CTL, in a similar manner to the CD group. We conducted a *t*-test on the left precuneus, and found the >100 use group showed significantly lower cortical thickness than CTL (*t* = 2.40, *p* = .020, Cohen’s *D* = .36) albeit the effects were not as strong as the CD vs. control comparison (Cohen’s *D* = .51) (**Supplementary Figure 3)**. This suggests a graded effect of cannabis use on precuneus structure.

**References**

1 Weiland BJ, Thayer RE, Depue BE, Sabbineni A, Bryan AD, Hutchison KE. Daily Marijuana Use Is Not Associated with Brain Morphometric Measures in Adolescents or Adults. *J Neurosci*  2015; **35**: 1505–1512.

2 Kaufman L, Rousseeuw PJ. *Finding Groups in Data: An Introduction to Cluster Analysis*. 1990.

3 Orr JM, Paschall CJ, Banich MT. Recreational marijuana use impacts white matter integrity and subcortical (but not cortical) morphometry. *NeuroImage Clin* 2016; **12**: 47–56.

4 Manza P, Tomasi D, Volkow ND. Subcortical local functional hyperconnectivity in cannabis dependence. *Biol Psychiatry Cogn Neurosci Neuroimaging* 2018; **3**: 285–293.

5 Arnatkevičiūtė A, Fulcher BD, Fornito A. A practical guide to linking brain-wide gene expression and neuroimaging data. *Neuroimage* 2019; **189**: 353–367.

6 Hawrylycz M, Miller JA, Menon V, Feng D, Dolbeare T, Guillozet-Bongaarts AL *et al.* Canonical genetic signatures of the adult human brain. *Nat Neurosci* 2015; **18**: 1832–1844.

7 Fulcher BD, Little MA, Jones NS. Highly comparative time-series analysis: the empirical structure of time series and their methods. *J R Soc Interface* 2013; **10**: 20130048.

8 Pagliaccio D, Barch DM, Bogdan R, Wood PK, Lynskey MT, Heath AC *et al.* Shared Predisposition in the Association Between Cannabis Use and Subcortical Brain Structure. *JAMA psychiatry* 2015; **72**: 994–1001.

9 Vainik U, Baker TB, Dadar M, Zeighami Y, Michaud A, Zhang Y *et al.* Neurobehavioural Correlates of Obesity are Largely Heritable. *Proc Natl Acad Sci* 2018; **115**: 9312–9317.

10 Bates D, Maechler M, Bolker B, Walker S. Fitting Linear Mixed-Effects Models Using lme4. *J Stat Softw* 2015. doi:10.18637/jss.v067.i01.

11 Lakens D. Equivalence Tests: A Practical Primer for t Tests, Correlations, and Meta-Analyses. *Soc Psychol Personal Sci* 2017; **8**: 355–362.

**Supplementary Table 1. Demographics and clinical characteristics for each group.** Note: CTL = Control (n = 89); CD = Cannabis Dependent (n = 89). The CTL group is well matched on all variables except for Tobacco usage and other illicit drug usage; therefore we tested whether any significant brain imaging results were associated with these variables in follow-up analyses.

|  | **CTL** | **CD** | **CD vs. CTL: *t* (*p*)** |
| --- | --- | --- | --- |
| Age | 28.6 ± 3.9 | 28.6 ± 3.5 | 0.02 (0.98) |
| BMI | 27.5 ± 5.2 | 26.8 ± 4.8 | -0.96 (0.34) |
| Education (Years) | 14.3 ± 1.8 | 14.3 ± 1.8 | -0.04 (0.97) |
| DSM Depression | 53.6 ± 5.5 | 54.3 ± 7.0 | 0.73 (0.47) |
| DSM Anxiety | 53.1 ± 5.1 | 53.7 ± 6.3 | 0.80 (0.43) |
| Alcohol (Composite Z) | 0.17 ± 0.42 | 0.22 ± 0.46 | 0.80 (0.43) |
| Tobacco (Composite Z) | -0.19 ± 0.56 | 0.79 ± 1.02 | 7.94 (<0.001) |
| Sex (% Male) | 64/89 (72%) | 64/89 (72%) |  |
| Used Illicit Drugs (other than cannabis) | 8/89 (9%) | 69/89 (78%) |  |
| Used cannabis in last 12 months | 5/89 (6%) | 62/89 (68%) |  |

**Supplementary Table 2. Participant Illicit Drug Use (other than cannabis).** Each cell represents the number (%) of participants within each group.

|  | **Never** | **1-2 Times** | **3-10 Times** | **11-25 Times** | **25-100 Times** | **>100 Times** | **Total N** |
| --- | --- | --- | --- | --- | --- | --- | --- |
| **CTL** | 81 (91.0) | 7 (7.9) | 1 (1.1) | 0 (0.0) | 0 (0.0) | 0 (0.0) | 89 |
| **CD** | 20 (22.5) | 4 (4.5) | 19 (21.3) | 11 (12.4) | 24 (27.0) | 11 (12.4) | 89 |
| **> 100 Use** | 38 (47.5) | 7 (8.8) | 16 (20.0) | 10 (12.5) | 6 (7.5) | 3 (3.8) | 80 |
| **All Others In HCP** | 805 (85.1) | 52 (5.5) | 49 (5.2) | 17 (1.8) | 20 (2.1) | 3 (0.3) | 946 |
| **Grand Total** | **944 (78.4)** | **70 (5.8)** | **85 (7.1)** | **38 (3.2)** | **50 (4.2)** | **17 (1.4)** | **1204** |

**Supplementary Table 3. Sibling-pair characteristics.** Each cell represents the number (%) of sibling pairs. Note: DZ = Dizygotic; MZ = Monozygotic

|  | **DZ or Non-Twin** | **MZ** | **Total N** |
| --- | --- | --- | --- |
| **Concordant, High Cannabis Exposure** | 30 (73.2) | 11 (26.8) | **41** |
| Both F | 7 (70.0) | 3 (30.0) | **10** |
| Both M | 18 (69.2) | 8 (30.8) | **26** |
| Opposite Sex | 5 (100.0) | 0 (0.0) | **5** |
|  |  |  |  |
| **Concordant, Low Cannabis Exposure** | 536 (82.8) | 111 (17.2) | **647** |
| Both F | 187 (71.1) | 76 (28.9) | **263** |
| Both M | 129 (78.7) | 35 (21.3) | **164** |
| Opposite Sex | 220 (100.0) | 0 (0.0) | **220** |
|  |  |  |  |
| **Discordant for Cannabis Exposure** | 119 (90.2) | 13 (9.8) | **132** |
| Both F | 27 (87.1) | 4 (12.9) | **31** |
| Both M | 35 (79.5) | 9 (20.5) | **44** |
| Opposite Sex | 57 (100.0) | 0 (0.0) | **57** |
|  |  |  |  |
| **Grand Total** | **685 (83.5)** | **135 (16.5)** | **820** |

**Supplementary Table 4. Contrast coding for the ‘Causal’, ‘Graded Liability’, and ‘Predisposition’ hypotheses.** These primary hypotheses were tested with a linear mixed model approach (see **Figure 2c** and **Supplementary Table 5**). Contrast 1 tested whether there may be causal effects of high cannabis exposure on precuneus cortical thickness, whereas contrasts 2 and 3 tested whether heavy cannabis use and precuneus cortical thickness may share predispositional factors; coding was adapted from Pagliaccio et al^8^.

|  | **Contrast 1:**  **‘Causal’ Hypothesis** | **Contrast 2:**  **‘Graded Liability’ Hypothesis** | **Contrast 3:**  **‘Predisposition’ Hypothesis** |
| --- | --- | --- | --- |
| Individual with Low Cannabis Exposure from Discordant Pair | -1 | -1 | -1 |
| Individual with High Cannabis Exposure from Discordant Pair | 1 | -1 | -1 |
| Concordant High Exposure Pairs | 0 | 2 | -1 |
| Concordant Low Exposure Pairs | 0 | 0 | 3 |

**Supplementary Table 5. Linear Mixed Model Results for Sibling Pairs: Effects of cannabis usage on left precuneus cortical thickness.** We included sex, age, alcohol usage, tobacco usage, BMI, income, education, depression and anxiety as covariates (i.e., the same covariates that were used to match the CD and CTL groups in the main manuscript). There was some support for ‘causal’ and ‘graded liability’ hypotheses, but not the ‘predisposition’ hypothesis (also see **Supplementary Table 4** for the contrasts, and **Fig. 2c** for the estimated marginal means in each group). Therefore, these data provide some preliminary evidence for a) causal effects of cannabis on precuneus cortical thickness, and b) that cortical thickness and heavy cannabis use might have common predispositional factors, with concordant exposed pairs at the highest liability (that is, ‘graded liability’).

|  | **Fixed Effect Estimate ± SEM** | ***t* (*p*)** |
| --- | --- | --- |
| Intercept | 2.5598 ± 0.0431 | 59.440 (0.000) |
| Sex (M>F) | -0.0014 ± 0.0057 | -0.240 (0.810) |
| Age | -0.0031 ± 0.0008 | -3.694 (0.000) |
| Alcohol (Composite Z-score) | -0.0102 ± 0.0056 | -1.809 (0.070) |
| Tobacco (Composite Z-score) | -0.0066 ± 0.0036 | -1.869 (0.062) |
| BMI | 0.0011 ± 0.0006 | 1.987 (0.047) |
| Income | -0.0013 ± 0.0013 | -0.926 (0.354) |
| Education | 0.0016 ± 0.0016 | 1.027 (0.304) |
| DSM Depression | 0.0002 ± 0.0006 | 0.287 (0.774) |
| DSM Anxiety | -0.0002 ± 0.0007 | -0.239 (0.811) |
| Contrast 1: 'Causal' | -0.0115 ± 0.0054 | -2.130 (0.033) |
| Contrast 2: 'Graded Liability' | -0.0102 ± 0.0050 | -2.039 (0.041) |
| Contrast 3: 'Predisposition' | 0.0030 ± 0.0022 | 1.378 (0.168) |

**Supplementary Figure 1. Association between group differences in cortical thickness and gray matter density**. Group differences in cortical thickness and gray matter density were generally consistent across brain regions; there was a positive correlation between the two (*r* = .53, *p* = .001). The solid line indicates the line of best fit, and the dashed lines indicate the 95% confidence intervals. Note: GM = gray matter.


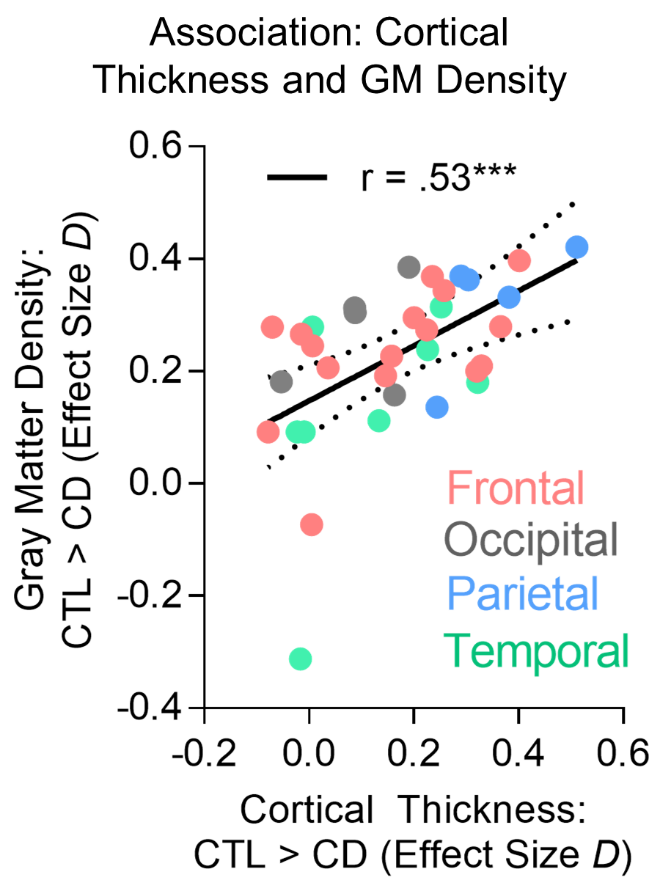


**Supplementary Figure 2. Precuneus cortical thickness based on past-year cannabis use and THC Urinalysis in the CD group.** Our results suggest that recent cannabis use, as assessed by a) subjective (self-reported number of cannabis uses in the past year) and b) objective (THC urine screen results) measures both suggested that recent use was not driving the precuneus thickness deficits in the CD group. Indeed, the primary results held when including these factors as covariates in the analyses. The numbers in each bar represent the number of participants. NOTE: L = Left. Error bars represent standard error of the mean.


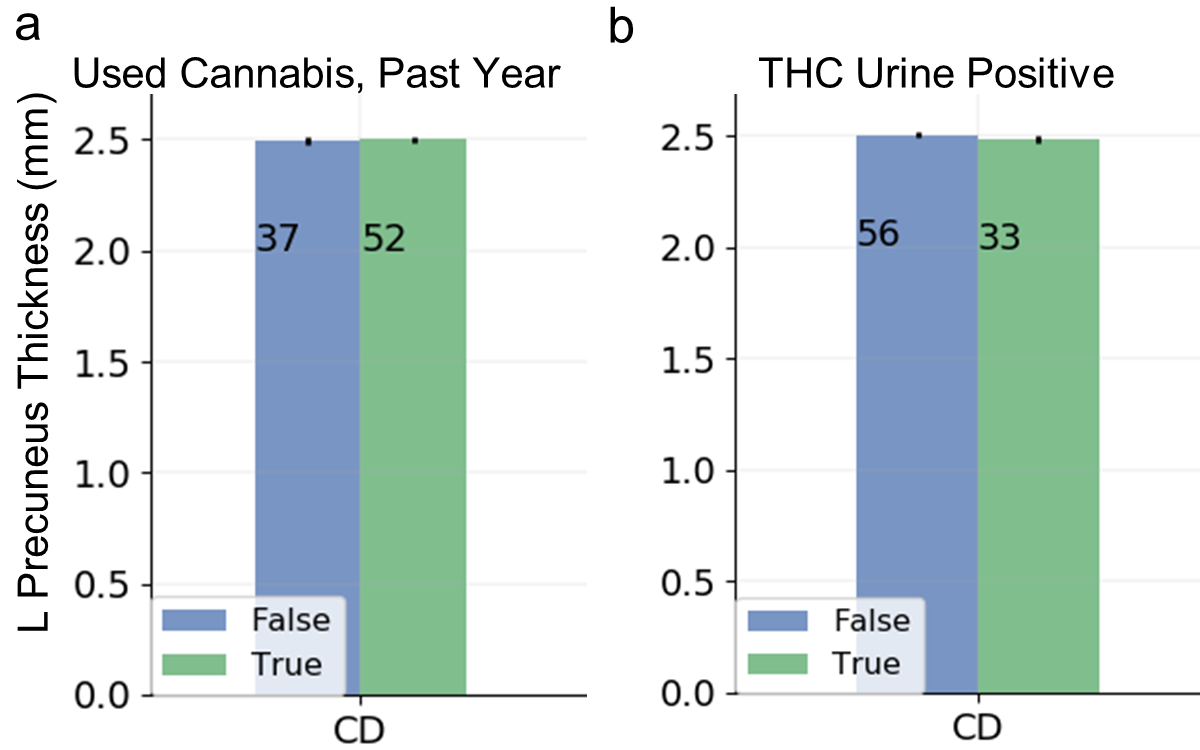


**Supplementary Figure 3. Group differences in left precuneus cortical thickness, including the ‘>100 Use’ group.** The >100 Use group showed significantly lower cortical thickness than CTL (*t* = 2.40, *p* = .020, Cohen’s *D* = .36) albeit the effects were not as strong as the CD vs. control comparison (Cohen’s *D* = .51). This suggests that there may be a graded effect of cannabis use on precuneus structure. Error bars represent standard error of the mean.


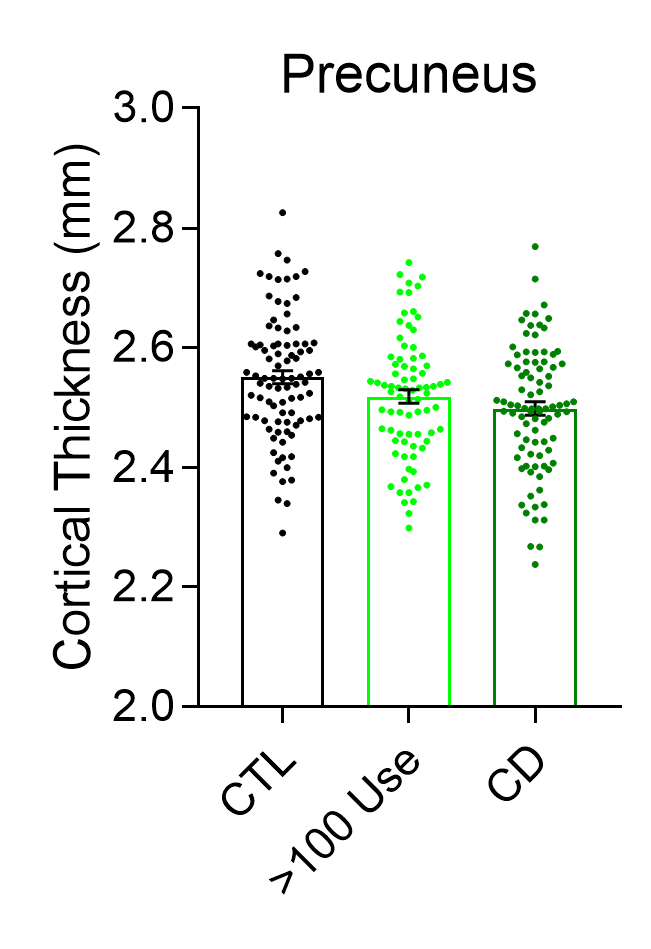

Supplement: Supplementary file 1 — Supplement [file 41380_2019_577_MOESM1_ESM.docx]
